# Association of plasma lactoferrin levels with disease severity in glaucoma patients

**DOI:** 10.3389/fmed.2024.1385358

**Published:** 2024-05-30

**Authors:** Zuo Wang, Donghua Liu, Hang Yuan, An Li, Jinxia Wang, Xiong Zhu, Wenbo Xiu, Gao Zhang, Yang Chen, Lingling Chen, Xiao Xiao, Chong He, Fang Lu

**Affiliations:** ^1^Department of Clinical Laboratory, Sichuan Clinical Research Center for Cancer, Sichuan Cancer Hospital & Institute, Sichuan Cancer Center, Affiliated Cancer Hospital of University of Electronic Science and Technology of China, Chengdu, China; ^2^Clinical Immunology Translational Medicine Key Laboratory of Sichuan Province, Sichuan Provincial People’s Hospital, University of Electronic Science and Technology of China, Chengdu, China; ^3^Department of Immunology, West China School of Basic Medical Sciences & Forensic Medicine, Sichuan University, Chengdu, China; ^4^Department of Prenatal Diagnosis, Chengdu Women’s and Children’s Central Hospital, School of Medicine, University of Electronic Science and Technology of China, Chengdu, China; ^5^Medico-Engineering Cooperation on Applied Medicine Research Center, University of Electronic Science and Technology of China, Chengdu, China; ^6^Health Management Center, Sichuan Provincial People’s Hospital, University of Electronic Science and Technology of China, Chengdu, China

**Keywords:** plasma lactoferrin, glaucoma, iron dysregulation, disease severity, pathogenic progression

## Abstract

**Objective:**

To explore the relationship between plasma lactoferrin (Lf) and glaucoma, assessing the clinical utility of Lf in glaucoma.

**Methods:**

A cross-sectional study involved 161 glaucoma patients and 115 healthy controls, with a follow-up of 14 subjects after approximately 2 years. Plasma Lf markers were quantified using ELISA, comparing levels between glaucoma patients and healthy controls, and analyzing plasma Lf across different glaucoma severity grades.

**Results:**

Glaucoma patients had significantly elevated plasma Lf levels compared to healthy controls (*p* < 0.001). Higher plasma Lf levels correlated with more severe disease stages (HPA grades showed *ρ* = 0.435, *p* < 0.001; AGIS grades showed *ρ* = 0.436, *p* < 0.001) and reduced retinal nerve fiber layer (RNFL) thickness (RNFL thickness showed *ρ* = −0.204, *p* = 0.024). ROC curve analysis demonstrated the efficacy of glaucoma markers in differentiating early-stage from advanced glaucoma.

**Conclusion:**

Plasma Lf levels are significantly associated with glaucoma severity and may be involved in the pathogenic progression of the disease.

## 1 Introduction

Glaucoma is a chronic, progressive and irreversible neurodegenerative disease and one of the major causes of blindness globally with characteristic loss of retinal ganglion cell (RGCs) and damage of the optic nerve (1). Glaucoma is a complex disease caused by multiple factors, the pathogenesis and exact mechanism have remained obscure and debatable. Among its numerous evidence-based risk factors, elevated intraocular pressure (IOP) has been recognized as the most critical. Recent studies have elucidated that heightened intraocular pressure disrupts the normal iron homeostasis, subsequently inducing ferroptosis in the retinal ganglion cells in glaucoma (2). In patients diagnosed with acute primary angle-closure glaucoma (APACG), the levels of serum total iron and ferric iron were higher than those in healthy control subjects (3). Besides, epidemiological investigations have demonstrated a positive correlation between a diet rich in iron and increased serum ferritin levels with a higher incidence of glaucoma (4–6). These findings indicate a close association between iron metabolism and the pathogenesis of glaucoma.

Lactoferrin (Lf), a member of the transferrin family, has been recognized as a new key regulator in iron metabolism and homeostasis (7). This iron-binding glycoprotein is predominantly found in biological fluids and neutrophil granules. Lf plays a crucial role in controlling intestinal iron absorption and in iron transport, and it has the capacity to chelate iron, both directly and indirectly. It facilitates iron binding and transfer through various receptors among cells, serum, bile, and cerebrospinal fluid, thereby playing an essential role in maintaining iron equilibrium. Lf is implicated in a variety of pathologies related to iron dysregulation (8). For example, Lf concentrations are notably elevated in individuals with Proliferative Diabetic Retinopathy (PDR) as compared to those in normal subjects (9). However, there are few studies exploring the association between plasma Lf and glaucoma.

The objective of this research is to elucidate the potential correlations between plasma Lf levels and glaucoma. We conducted a comparative analysis of plasma Lf levels in glaucoma patients versus healthy controls, further examining these levels in correlation with varying degrees of glaucoma severity. Additionally, we assessed the relationship between plasma Lf levels and patient stratification based on retinal nerve fiber layer (RNFL) thickness and vertical cup-to-disc ratio (VCDR). Our objective was to find the potential clinical significance of plasma Lf in glaucoma.

## 2 Materials and methods

### 2.1 Subjects

Our study enrolled 161 patients, all of whom were enrolled from the Sichuan Provincial People’s Hospital. Adhering strictly to the Declaration of Helsinki guidelines, this study received full approval from the Institutional Review Board for Clinical Research at the Sichuan Provincial People’s Hospital (No. 201968). All individuals who are hospitalized to Sichuan Provincial People’s Hospital provided their written, informed permission for the use of their clinical data in this study. Between January 2021 and July 2023, comprehensive information was acquired from the medical records of the patients. As previously documented, ophthalmologists with specialized expertise employed ophthalmic examinations in conjunction with factors such as age, family history, and clinical manifestations indicative of glaucoma to establish a diagnosis. The following ocular tests were performed on each participant: IOP, mean deviation (MD), vertical cup-to-disk ratio (VCDR), thickness of the retinal nerve fiber layer (RNFL), and VF loss. To determine IOP, Goldmann applanation tonometry was employed. MD, RFNL, and VCDR were measured using OCT. VF was measured using automated perimetry as is customary. The following conditions had to be met in order for glaucoma patients to be included in the study: they had to be clinically diagnosed with the condition and be free of any autoimmune, inflammatory, or neurodegenerative illnesses (such as AD or PD). Additionally, this study involved an approximately two-year follow-up of 14 glaucoma patients, during which a series of ocular assessments were conducted on each individual. A total of 115 healthy controls who were matched for age and gender were included. The following were included as exclusion criteria: having glaucoma or a family history of glaucoma, complaining of eye pain, having an elevated IOP (>21 mmHg), having undergone recent surgery, or having any other neurological illnesses.

### 2.2 Collection of blood samples and ELISA

After patients had fasted for 8 h, blood samples were taken in the morning from the anterior elbow veins using EDTA-anticoagulated Vacutainer CPT tubes. Plasma was collected by centrifuging tubes for 10 min at 3,000 revolutions per minute. The plasma Lf level was determined using commercially Human LF ELISA kits (FineTest Batch No: FN230925). The experiment was carried out exactly as instructed.

### 2.3 Determination of glaucoma severity

Two methods were employed to ascertain the severity of glaucoma: the Hodapp, Parish, and Anderson (H-P-A) classification system and the advanced glaucoma intervention study scoring (AGIS) system (10). The H-P-A classification system primarily relied on the mean deviation (MD) of the visual field to determine the severity of glaucoma. An MD of greater than −6 dB indicated an early stage, while an MD ranging from −12 dB to −6 dB denoted a moderate stage, and an MD of no greater than −12 dB indicated a severe stage. Additional criteria for mild glaucoma stipulate that all points within the central 5 degrees must exhibit a sensitivity of at least 15 dB. Conversely, criteria for severe glaucoma require that points within the central 5 degrees demonstrate a sensitivity of less than 15 dB. On the other hand, the AGIS system determined the severity of glaucoma based on VF scores. A visual field score ranging from 3 to 5 indicated an early stage, a score ranging from 6 to 12 represented a moderate stage, a score ranging from 13 to 18 indicated a severe stage, and a score ranging from 18 to 20 was classified as end-stage glaucoma. Points were assigned to the score according to the following criteria: (1) If there was a nasal defect or nasal step, one point was added to the score. Additionally, if four or more of the six nasal test locations exhibited a depression of 12 dB or more, an additional point was added to the score. (2) In each hemifield, if there were one or more clusters of three or more adjacent depressed test locations (hemifield defects), one point was added to the score if there were 3 to 5 depressed test sites in the clusters; two points were added if there were 6 to 12 depressed test sites; three points were added if there were 13 to 20 depressed test sites; and four points were added if there were more than 20 depressed test sites. (3) If half or more of the adjacent defective locations in a hemifield exhibited a depression of 28 dB or more, five points were added to the score. Similarly, if half or more of the adjacent defective locations were depressed 24 dB or more, four points were added; if depressed 20 dB or more, three points were added; if depressed 16 dB or more, two points were added; and if depressed 12 dB or more, one point was added. This series of steps could potentially add up to five points to the score for each hemifield containing a deep defect. (4) If a hemifield lacked a cluster of three adjacent depressed test sites but contained at least two adjacent depressed sites, with one site having a depression of 12 dB or more, one point was added to the score. (5) The scores for each hemifield and for the nasal area were then summed. The maximum achievable score was 20, with 2 points for the nasal field and 9 points for each hemifield.

### 2.4 Statistics analysis

For the statistical study, R (version 4.1.3) was utilized. The Kolmogorov–Smirnov test was performed to determine the normality of the distribution. For properly distributed data, the unpaired student’s *t*-test (two-tailed) was performed. For non-parametric statistical testing, the Mann–Whitney *U* test was utilized. For non-normally distributed paired comparisons of clustered data, the Wilcoxon signed-ranks test was utilized. For categorical data analysis, the chi-square test was performed. The study employs Spearman’s rank correlation coefficient for the analysis of correlations. The diagnostic capabilities were determined using receiver operating characteristics (ROC) curve analysis and the computation of the area under the ROC curve (AUC). *p* < 0.05 was regarded as statistically significant.

## 3 Results

### 3.1 Demographics of the participants

The demographics of these participants are shown in Table 1. Our study included 161 patients affected by glaucoma and 115 healthy controls who were age and gender matched. The glaucoma group showed a mean age of 54.73 years while the control group showed a mean age of 51.99 years (*p* = 0.07). In the glaucoma group, there were 75 men (46.58%) and 62 (53.91%) in the control group (*p* = 0.8213). we categorized patients with glaucoma into 3 (early, *n* = 40; moderate, *n* = 26; severe, *n* = 89) and 4 subgroups (early, *n* = 55; moderate, *n* = 33; severe, *n* = 9; end-stage: *n* = 57) each based on their severity of damage using the HPA classification system and the AGIS system.

**Table 1:** Demographics of glaucoma patients and healthy controls.

Variable	Glaucoma	Healthy controls	*p*-value
Age (years)	54.73 ± 11.64	51.99 ± 12.95	0.07
Male/female	75/86	62/53	0.23
HPA stages: early/moderate/severe	40/26/89	/	/
AGIS stages: early/moderate/severe/end-stage	55/33/9/57	/	/

Age is presented as mean ± SD. H-P-A, Hodapp, Parish, and Anderson; AGIS, advanced glaucoma intervention study scoring. The difference of age between glaucoma patients and healthy controls were examined by unpaired student’s *t*-test (two-tailed) and the difference of gender was examined by chi-square test. *p* < 0.05 was considered statistically significant.

### 3.2 Increased plasma Lf level in glaucoma patients

**Figure 1:**
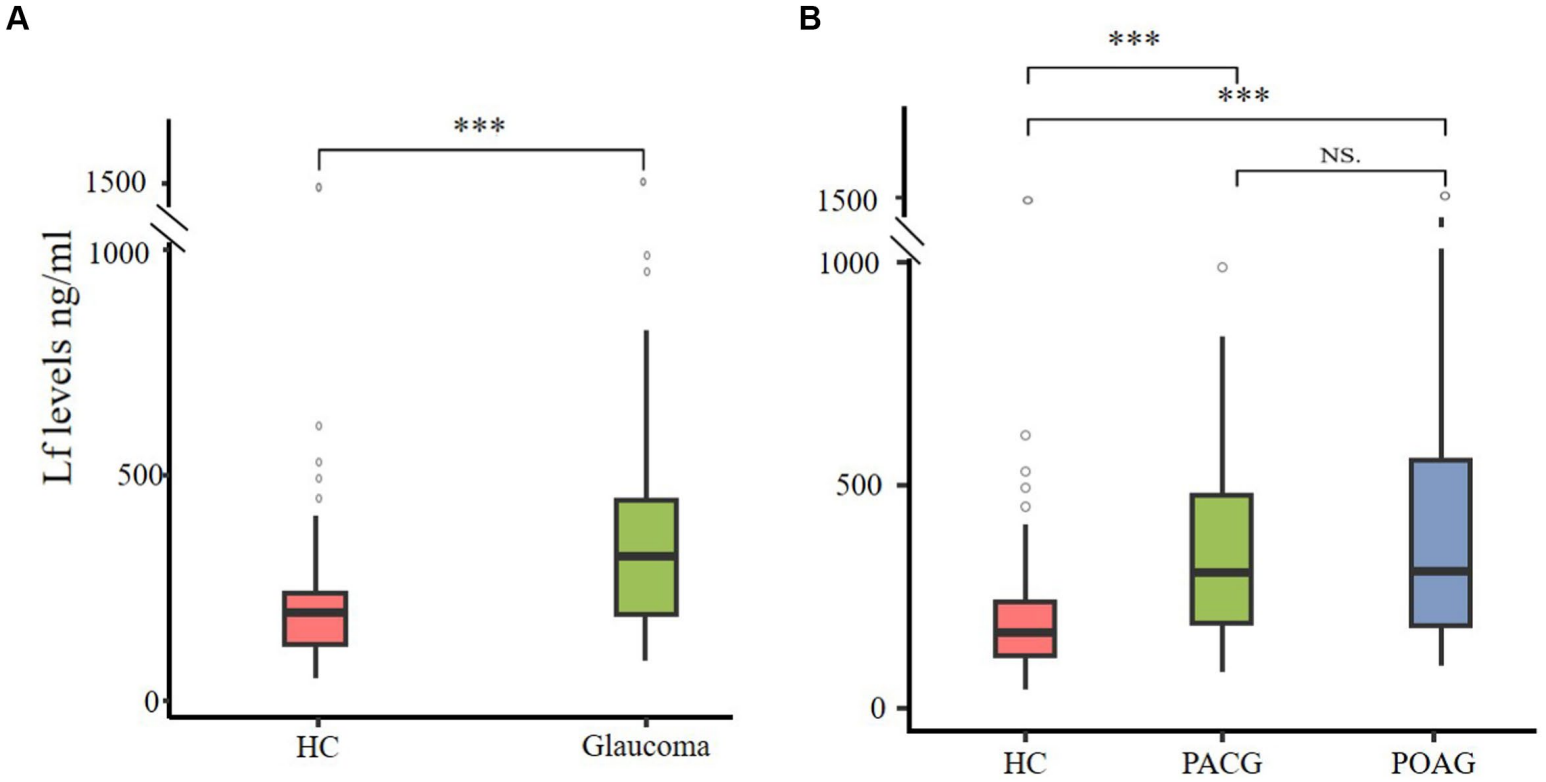
Plasma Lf levels in glaucoma patients. **(A)** Elevated plasma Lf in glaucoma patients. **(B)** Comparing plasma Lf levels among healthy controls (HC), primary angle-closure glaucoma (PACG) and primary open-angle glaucoma (POAG) subgroups. Group differences were evaluated using the Mann–Whitney *U* test for between-group comparisons, and the Kruskal–Wallis test was utilized to determine variations among primary angle-closure glaucoma (PACG), primary open-angle glaucoma (POAG), and healthy controls. NS (not significant) indicates no statistical significance; ^∗∗∗^*p* < 0.001.

This study aimed to examine the variations in plasma Lf levels in glaucoma. We compared the prevalence of Lf between glaucoma patients and a cohort of healthy controls. The findings revealed a statistically significant elevation in plasma Lf levels among patients with glaucoma in contrast to the healthy control group (Figure 1A). Among the various glaucoma types, primary open-angle glaucoma (POAG) and primary angle-closure glaucoma (PACG) are the most common. In our study, we compared plasma Lf levels in PACG and POAG groups with those in healthy controls. The median plasma Lf levels were significantly higher in both PACG, and POAG groups compared to the control group. However, there was no significant difference in plasma Lf levels between the POAG and PACG groups (Figure 1B).

### 3.3 The association between plasma Lf level and glaucoma neural damage and glaucoma severity

**Figure 2:**
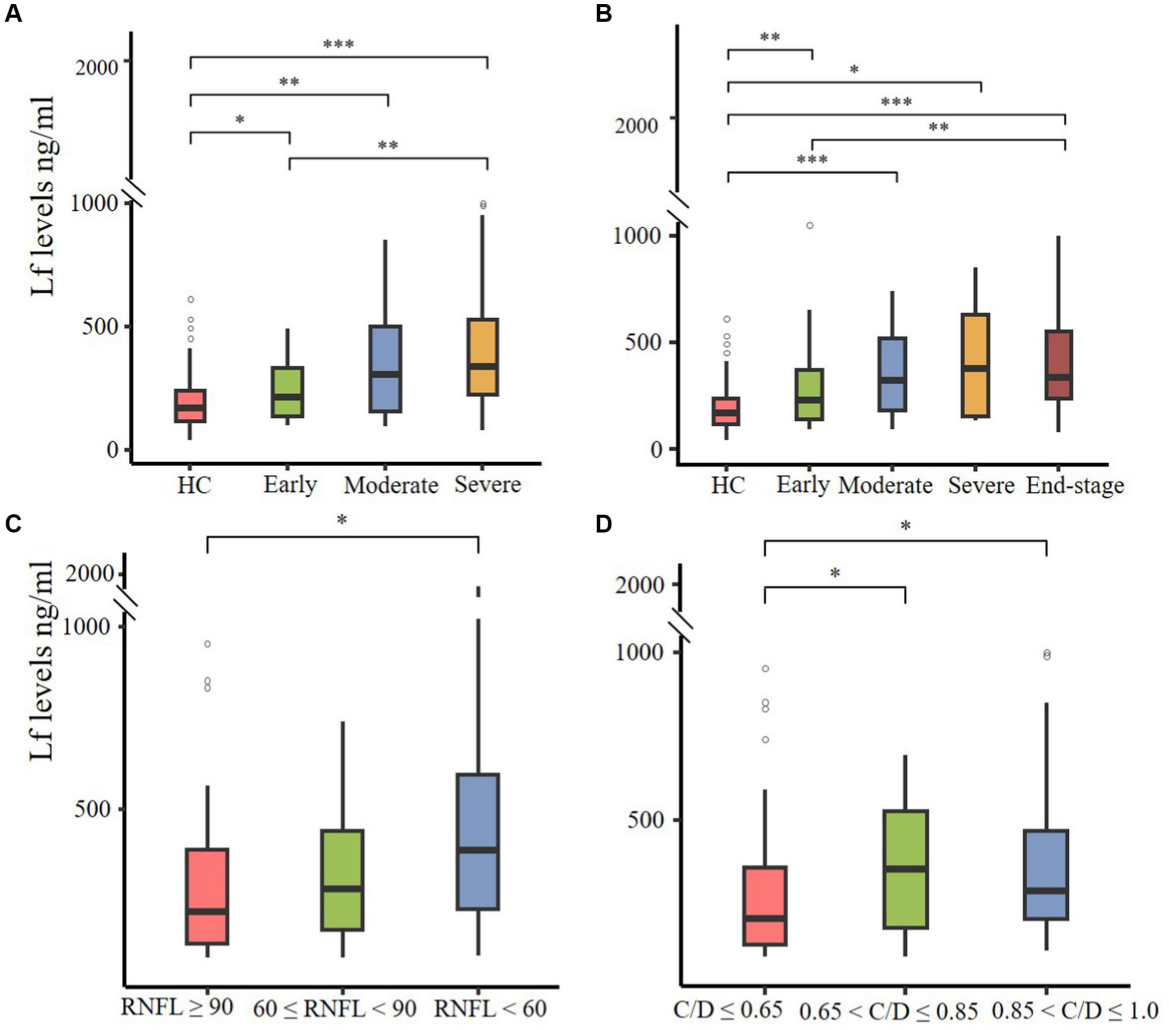
The correlation between plasma Lf levels, glaucoma severity, and optic nerve damage. **(A,B)** Plasma Lf levels in various glaucoma patient groups, stratified by disease severity using the Hodapp–Parrish–Anderson (H-P-A) classification system **(A)** and the advanced glaucoma intervention study (AGIS) system **(B)**. **(C,D)** Plasma Lf levels were evaluated in groups stratified by retinal nerve fiber layer (RNFL) thickness **(C)** and vertical cup-to-disc ratio (C/D) **(D)**. The differences in all parameters were assessed using the unpaired Wilcoxon rank-sum test. Significance levels are indicated as ^*^p < 0.05, ^**^p < 0.01 and, ^***^p < 0.001.

Then we utilized two disease staging systems to compare the plasma Lf level among patients with different disease severity in glaucoma. Based on the H-P-A classification system and the AGIS system, patients were categorized into three (early, moderate, and severe) and four (early, moderate, severe, and end-stage) subgroups. The severity of glaucoma was associated with an increased level of Lf in both classification systems, which suggests its potential use in discriminating advanced stage from early stage. Notably, patients with different disease severity levels differed in the amount of LF compared to healthy controls according to the H-P-A classification system (Figure 2A) and the AGIS method (Figure 2B), At the same time, we grouped patients based on their RNFL thickness and VCDR to examine the correlation between plasma Lf levels and optic nerve damage (11, 12). According to the findings, RNFL thinning (Figure 2C) and VCDR increasing (Figure 2D) were associated with an increase in LF levels. Lf levels were unaffected by the IOP (Supplementary Figure S1A), age (Supplementary Figure S1B), or gender (Supplementary Figure S1C).

To further elucidate the relationship between plasma lactoferrin (Lf) levels and glaucoma severity, we conducted Spearman correlation analyses using various indicators: HPA, AGIS scores, RNFL thickness, and VCDR. The results indicated significant correlations: HPA grades showed *ρ* = 0.435, *p* < 0.001; AGIS grades showed *ρ* = 0.436, *p* < 0.001; RNFL thickness showed *ρ* = −0.204, *p* = 0.024; and VCDR showed *ρ* = 0.127, *p* = 0.145 (no statistical significance). These findings suggest a positive association between higher plasma Lf levels and increased severity of glaucoma, as well as greater neural damage.

To evaluate the association between plasma Lf levels and the dynamic progression of glaucoma, we assembled a cohort of 14 subjects prior to their undergoing ocular hypotensive surgery. Over a follow-up period of approximately two years, we analyzed ophthalmological examination data, using a decline in retinal nerve fiber layer (RNFL) thickness of −2 μm/year as an indicator of glaucoma progression (13). We established a cutoff at 477 ng/mL for plasma Lf levels to correlate with changes in RNFL thickness. Our findings revealed that 6 out of 14 patients exhibited abnormally high baseline plasma Lf levels (>477 ng/mL), while the remaining 8 patients had normal or elevated baseline plasma Lf levels (<477 ng/mL). During the study, 5 of the 6 subjects with high baseline plasma Lf levels demonstrated a reduction in RNFL thickness. In contrast, only 1 of the 8 subjects with normal or elevated baseline plasma Lf levels showed a decrease in RNFL thickness (*p* = 0.026, Fisher’s exact test).

### 3.4 Analysis of receiver operating characteristic curves

ROC curves were generated, and the area under the curve (AUC) was measured to evaluate Lf ability to distinguish healthy controls from glaucoma patients (AUC = 0.738, Sensitivity = 68.9%, Specificity = 71.3%). We then assessed disease severity degrees. The AUC of Lf in the H-P-A classification system was 0.630, 0.703, and 0.787 for identifying healthy controls from patients with early, moderate, and severe glaucoma, respectively (Table 2). In the advanced glaucoma intervention study (AGIS) system, the AUC for Lf was 0.651, 0.738, 0.727, and 0.808 for differentiating healthy controls from patients with early, moderate, severe, and end-stage glaucoma, respectively (Table 2). The results showed an increase in LF’s discriminative ability with escalating glaucoma severity. Further analysis revealed Lf’s efficacy in differentiating between early and advanced stages of glaucoma. As indicated in Table 2, Lf demonstrated satisfactory precision (early vs. severe: AUC = 0.665, Youden index = 0.318; early vs. end-stage: AUC = 0.670, Youden index = 0.299, according to the H-P-A and AGIS systems, respectively) in distinguishing between early and advanced glaucoma, suggesting its utility in identifying disease severity.

**Table 2:** Independent diagnostic value of plasma Lf in different stages of glaucoma.

		AUC	Cutoff (ng/mL)	Specificity (%)	Sensitivity (%)	Max. Youden
HPA	Early vs. HC	0.630	215.148	52.5	71.3	0.238
	Moderate vs. HC	0.703	273.987	61.5	81.7	0.432
	Severe vs. HC	0.787	216.587	76.4	71.3	0.477
	Early vs. Severe	0.665	289.563	70.0	61.8	0.318
AGIS	Early vs. HC	0.651	217.484	56.4	71.3	0.277
	Moderate vs. HC	0.738	215.148	67.6	71.3	0.389
	Severe vs. HC	0.727	378.352	55.6	94.8	0.504
	End-stage vs. HC	0.808	220.283	80.7	71.3	0.520
	Early vs. End-stage	0.670	232.656	77.2	52.7	0.299

Glaucoma severity was classified using the Hodapp–Parrish–Anderson (H-P-A) and advanced glaucoma intervention study (AGIS) systems. receiver operating characteristic (ROC) curve analysis was conducted, and the maximum Youden index was determined, calculated as sensitivity plus specificity minus one.

## 4 Discussion

Iron metabolism is a complex and intricately co-regulated process, mediated by a network of essential proteins including transferrin, ferritin, and iron responsive element-binding proteins (14). Within this network, lactoferrin, a member of the transferrin family, has been identified as a crucial regulator in iron metabolism and homeostasis, demonstrating a capacity to mitigate iron-related disorders across various pathologies (7, 8). Recent studies have highlighted the role of disrupted iron homeostasis in the pathogenesis of glaucoma (3, 15, 16). *In vitro* studies have demonstrated that LF can traverse the blood–brain barrier (BBB) as an intact protein via LfR-mediated transcytosis (17). The retina is regarded as an extension of the central nervous system (CNS). Its neurodegenerative processes and immune responses exhibit significant parallels to those observed in Parkinson’s disease (PD) and Alzheimer’s disease (AD). In PD, immunohistochemical analysis of post-mortem brain tissues has demonstrated elevated Lf expression in PD patients compared to control subjects (18). In AD, there is a notable upregulation of Lf in the cerebral tissues, implicating its function as an iron scavenger. This upregulation likely signifies a defensive response in AD-afflicted brain tissue (19). Correspondingly, our research reveals heightened plasma Lf levels in individuals with glaucoma, which may indicate Lf’s involvement in mitigating iron-associated pathologies via iron chelation in this condition.

Lf is pivotal not only in regulating iron metabolism but also in exerting antioxidant effects. Notably, elevated serum ferritin levels, indicative of higher iron stores, were significantly observed in patients with primary open-angle glaucoma (POAG) compared to healthy controls, alongside a markedly reduced total iron binding capacity (5). Additionally, another study underscores the association between increased serum ferritin levels and an elevated risk of glaucoma in the South Korean population (6). Ferritin served as a surrogate indicator of the body’s iron stores. Therefore, the elevated ferritin suggests iron overload in glaucoma. Iron overload exacerbates retinal pathologies through mechanisms such as oxidative stress, inflammation, and cellular apoptosis. Excess tissue iron, a potent facilitator of free radical generation, markedly heightens the risk of degenerative diseases like Parkinson’s, Alzheimer’s, and glaucoma (17). Recent research has increasingly acknowledged the significant role of oxidative stress in the pathogenesis of glaucoma (20). Oxidative stress, associated with factors such as elevated intraocular pressure and aging, is implicated in the progression of glaucoma, offering insights into the mechanisms underlying these risk factors. The antioxidant properties of Lf are predominantly attributed to its iron-binding capacity, which reduces the generation of reactive oxygen species (ROS) (8). Additionally, Lf acts as a ROS scavenger, thereby protecting DNA from oxidative damage. Consequently, Lf may play a role as an antioxidant in the pathogenesis of glaucoma.

Recent evidence highlights systemic inflammation’s potential role in glaucoma pathogenesis. Complete blood count (CBC) indicators such as neutrophil count, neutrophil-to-lymphocyte ratio (NLR), platelet-to-lymphocyte ratio (PLR), lymphocyte-to-monocyte ratio (LMR), and systemic immune-inflammation index (SII) may serve as inflammatory biomarkers in various diseases. Notably, NLR, PLR, and SII values were significantly higher in the POAG group compared to controls, with NLR and SII escalating with disease severity (21). PLR was notably higher in the progression group than in the non-progression group, demonstrating a significant correlation between elevated PLR and heightened risk of VF loss progression in glaucoma patients (22). In our previous study, parameters including neutrophil-to-albumin ratio (NAR), neutrophil-to-total bilirubin ratio (NTBR), and neutrophil-to-indirect bilirubin ratio (NIBR) were significantly higher in glaucoma patients compared to healthy controls, showing a positive correlation with clinical visual impairment (23). Additionally, local inflammatory processes in the retinal tissue have been identified, extending beyond systemic inflammation. Research using the chronic ocular hypertension glaucoma model and DBA/2J mice model demonstrated significant elevations of pro-inflammatory cytokines IL-6 and IL-1β in the retina (24). Emerging evidence indicates that elevated plasma or serum Lf levels are linked to the development of inflammatory diseases, exhibiting anti-inflammatory and immunomodulatory effects. Lf significantly reduces pro-inflammatory cytokines IL-6, IL-8, and IL-1β (25). In pediatric inflammatory bowel disease (IBD) patients, characterized by recurrent, persistent intestinal inflammation, Lf levels were significantly higher than in healthy controls (26). Similar elevations in Lf were observed in serum or plasma of patients with rheumatoid arthritis (RA) (27). Consequently, Lf’s anti-inflammatory properties might also play a role in glaucoma progression.

Advanced glaucoma is a major risk factor for blindness. Diagnosis and monitoring traditionally depend on structural and functional assessments, including IOP, OCT, and standard automatic perimetry. However, these methods can be impractical for some patients due to their time-consuming and costly nature, coupled with the coordination required during examinations, particularly challenging for elderly patients. Fortunately, Recent studies have established a notable link between peripheral blood biomarkers and the severity and progression of visual field (VF) loss in glaucoma patients (21, 22). Given the observed differences in Lf levels between glaucoma patients and healthy controls, alongside its simplicity, cost-effectiveness, and accessibility as a peripheral blood biomarker, we further explored Lf levels in patients with varying stages of glaucoma and in healthy individuals. This highlights Lf’s potential as a novel tool for glaucoma detection, screening, and severity assessment. Notably, Lf exhibited high diagnostic accuracy in distinguishing between early and advanced stages of glaucoma, as well as between healthy individuals and patients with severe glaucoma. Additionally, other studies across various diseases have linked plasma Lf levels with disease activity and severity; for instance, in severe sepsis in children, high plasma Lf concentrations were associated with the onset of organ failure. Furthermore, Lf has been reported to correlate with the severity of PD (28). Therefore, Plasma Lf levels are indicative of glaucoma severity and may be implicated in the disease’s pathogenic progression.

Our study has several limitations. First, while Lf shows promise in differentiating glaucoma patients from healthy controls, future research with larger sample sizes is needed to enhance its diagnostic accuracy. Second, our study’s design, combining cross-sectional case–control with follow-up, did not explore the potential mechanisms linking Lf and glaucoma, which will be a primary focus in subsequent research. Third, the follow-up phase included a limited number of glaucoma patients, indicating a need for more comprehensive, long-term longitudinal studies. Finally, as this study was conducted at a single center, further multicenter research across diverse ethnic groups is essential to validate our findings.

In conclusion, this study is the initial evaluation of plasma lactoferrin in glaucoma, revealing its potential clinical value in reflecting the disease’s severity.

## Data availability statement

The original contributions presented in the study are included in the article/Supplementary material, further inquiries can be directed to the corresponding authors.

## Ethics statement

The studies involving humans were approved by the Sichuan Provincial People’s Hospital. The studies were conducted in accordance with the local legislation and institutional requirements. The human samples used in this study were acquired from gifted from another research group. Written informed consent for participation was not required from the participants or the participants’ legal guardians/next of kin in accordance with the national legislation and institutional requirements.

## Author contributions

ZW: Conceptualization, Data curation, Formal analysis, Funding acquisition, Investigation, Methodology, Project administration, Resources, Software, Supervision, Validation, Visualization, Writing – original draft, Writing – review & editing. DL: Conceptualization, Data curation, Formal analysis, Investigation, Methodology, Project administration, Resources, Software, Supervision, Validation, Visualization, Writing – original draft, Writing – review & editing. HY: Data curation, Formal analysis, Investigation, Methodology, Validation, Visualization, Writing – review & editing. AL: Data curation, Formal analysis, Investigation, Methodology, Supervision, Validation, Visualization, Writing – review & editing. JW: Data curation, Formal analysis, Investigation, Methodology, Supervision, Validation, Writing – review & editing. XZ: Data curation, Formal analysis, Investigation, Methodology, Writing – review & editing. WX: Data curation, Investigation, Methodology, Supervision, Validation, Writing – review & editing. GZ: Data curation, Investigation, Methodology, Supervision, Validation, Writing – review & editing. YC: Data curation, Investigation, Methodology, Supervision, Writing – review & editing. LC: Data curation, Investigation, Methodology, Supervision, Writing – review & editing. XX: Data curation, Investigation, Methodology, Supervision, Writing – review & editing. CH: Conceptualization, Data curation, Formal analysis, Funding acquisition, Investigation, Methodology, Project administration, Resources, Software, Supervision, Validation, Visualization, Writing – original draft, Writing – review & editing. FL: Conceptualization, Data curation, Formal analysis, Funding acquisition, Investigation, Methodology, Project administration, Resources, Software, Supervision, Validation, Visualization, Writing – original draft, Writing – review & editing.

## Supplementary material

SUPPLEMENTARY FIGURE S1 Correlation of plasma Lf and glaucoma. **(A)** Comparisons of plasma Lf levels were conducted between male and female patients. Correlation analysis of plasma Lf with **(B)** intraocular pressure (IOP) and **(C)** age in glaucoma patient and spearman correlation *r* values were displayed. Statistical comparisons were performed using **(A)** Mann–Whitney test; **(B,C)** Spearman correlation.
