# Supplementary figures and images for: Association of plasma lactoferrin levels with disease severity in glaucoma patients
Source: Front Med (Lausanne). 2024 May 30;11:1385358. doi: 10.3389/fmed.2024.1385358 (PMC11169593; doi:10.3389/fmed.2024.1385358)

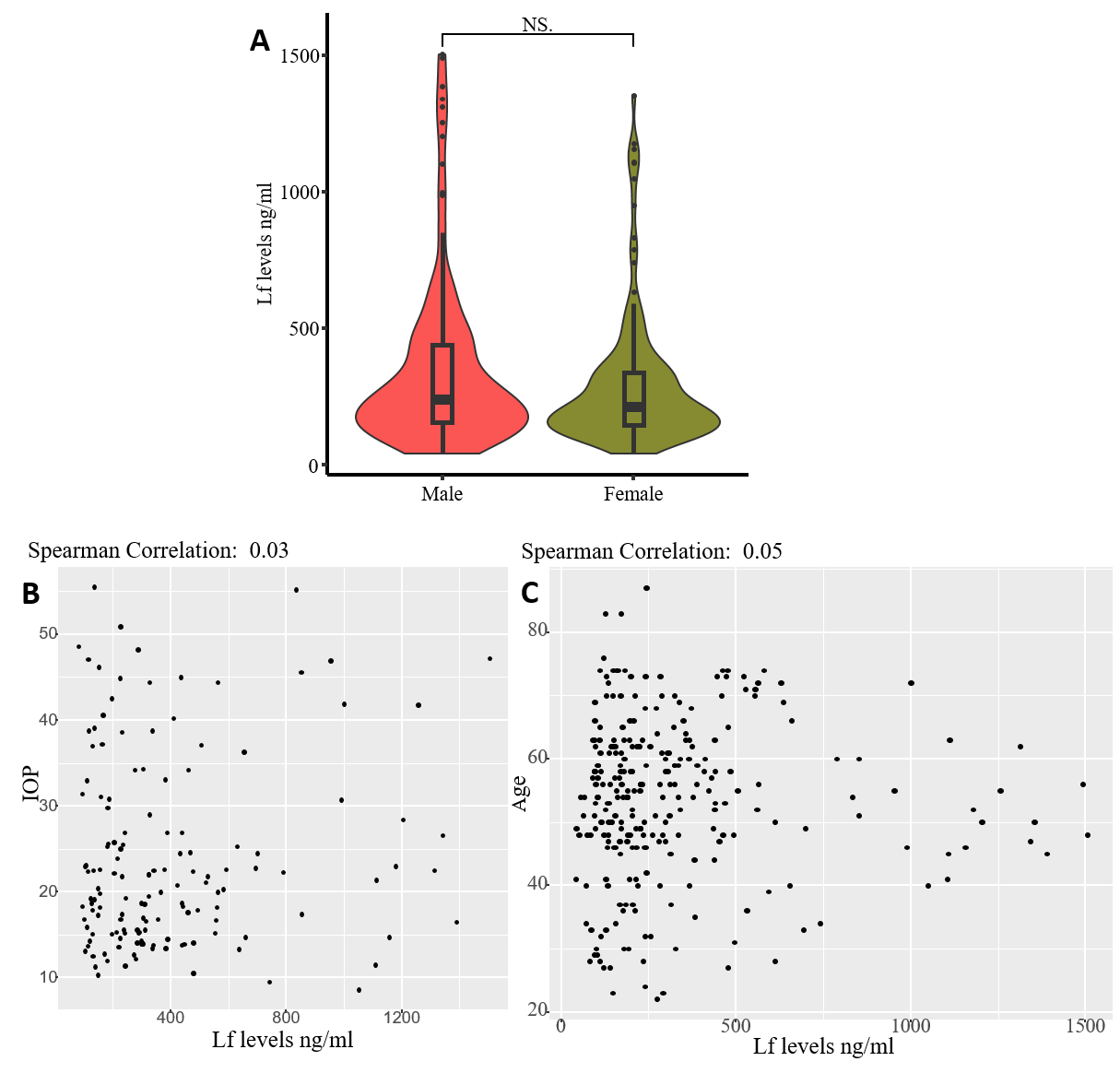

Supplement: SUPPLEMENTARY FIGURE S1 — Correlation of plasma Lf and glaucoma. (A) Comparisons of plasma Lf levels were conducted between male and female patients. Correlation analysis of plasma Lf with (B) intraocular pressure (IOP) and (C) age in glaucoma patient and spearman correlation r values were displayed. Statistical comparisons were performed using (A) Mann–Whitney test; (B,C) Spearman correlation. [file Image_1.TIF]
